# LNK promotes the growth and metastasis of triple negative breast cancer via activating JAK/STAT3 and ERK1/2 pathway

**DOI:** 10.1186/s12935-020-01197-9

**Published:** 2020-04-15

**Authors:** Jianxin Lv, Wei Yu, Yanan Zhang, Xinhua Cao, Lifei Han, Haolin Hu, Chenfei Wang

**Affiliations:** 1 Breast Disease Center, Zhongda Hospital, School of Medicine, Southeast University, Nanjing, China; 2 Jiangsu Key Laboratory of Drug Screening, China Pharmaceutical University, Nanjing, China

**Keywords:** LNK, Triple-negative breast cancer, p-ERK, JAK/STAT3

## Abstract

**Background:**

LNK adaptor protein is a crucial regulator of normal hematopoiesis, which down-regulates activated tyrosine kinases at the cell surface resulting in an antitumor effect. To date, little studies have examined activities of LNK in solid tumors except ovarian cancer.

**Methods:**

Clinical tissue chips were obtained from 16 clinical patients after surgery. Western blotting assay and quantitative real time PCR was performed to measure the expression of LNK. We investigate the in vivo and vitro effect of LNK in Triple Negative Breast Cancer by using cell proliferation、migration assays and an in vivo murine xenograft model. Western blotting assay was performed to investigate the mechanism of LNK in triple negative breast cancer.

**Results:**

We found that the levels of LNK expression were elevated in high grade triple-negative breast cancer through Clinical tissue chips. Remarkably, overexpression of LNK can promote breast cancer cell proliferation and migration in vivo and vitro, while silencing of LNK show the opposite phenomenon. We also found that LNK can promote breast cancer cell to proliferate and migrate via activating JAK/STAT3 and ERK1/2 pathway.

**Conclusions:**

Our results suggest that the adaptor protein LNK acts as a positive signal transduction modulator in TNBC.

## Background

Breast cancer is one of the most high incidence and mortality rate disease in the world [1], which is a heterogeneous disease and there are multiple ways by which to classify breast cancer into its subtypes [2]. Clinically, the primary diagnosis remains the histopathology report of the tumor which assesses the presence or absence of hormone receptors for estrogen (ER), progesterone (PR), and the human epidermal growth factor receptor-2 (HER2) [3]. The expression of these receptors is required to determine the patient’s suitability for endocrine therapies such as Tamoxifen, Anastrozole, and Trastuzumab [4]. The majority of breast cancers are receptor positive (77%) [5] and targeted treatment has proven efficacy. However, in the case of breast cancers that are negative for all three receptors (triple negative breast cancers, TNBC) there is, as yet, no targeted treatment available. Therefore, TNBC is more difficult to treat than target-specific breast cancer in clinical treatment [6–8]. And the only available treatments are chemotherapy and surgery [9]. Until now, numerous of trials with PARP inhibitors, angiogenesis inhibitors, EGFR inhibitors, SRC kinase inhibitors, and androgen receptor inhibitors have been used for therapy of TNBC, but none of them displayed significant improvements in all TNBC cases because of the heterogeneity of disease [9, 10]. Therefore, TNBC has a poor prognosis, which is associated with an increased number and earlier appearance of metastases (on average within the first 2.6 years after diagnosis) compared to other breast cancer subtypes [6, 9, 10]. Therefore, it is urgently to explore the therapeutic targets and new drugs of TNBC.

The lymphocyte adaptor protein LNK (SH2B3) is a key negative regulator of the signaling pathway of hematopoietic receptors activated by growth factors, thus playing a critical role in hematopoiesis [11–13] . LNK contains a N-terminal proline-rich region which mediates dimerization, a pleckstrin homology (PH) domain and a SRC homology 2 (SH2) domain which specifically binds to phosphorylated tyrosines and mediates signal transduction [14, 15]. LNK participates in many major signaling pathways, including those related to interleukin (IL)-3, stem cell factor (SCF)/c-Kit, thrombopoietin (TPO)/myeloproliferative leukemia protein (MPL), erythropoietin (EPO)/EPO receptor (EPOR), platelet-derived growth factor (PDGF)/PDGF receptor (PDGFR), tumor necrosis factor (TNF), and integrin [16, 17]. Previous studies indicated that overexpression of LNK activated the transduction of the mitogenic signal [18, 19]. Recent studies showed that LNK mutations have also been found in patients with myeloproliferative neoplasms (MPN) [20] and mainly mutated in hematopoietic malignancies including 3–5% of MPN samples, 10% of MPN evolved to acute myeloid leukemia, and 5% of early T cell leukemia [21]. Other studies showed that LNK mainly mutated in hematological and non-hematological malignancies, Acute lymphoblastic leukemia, Myeloproliferative neoplasms [13, 19, 22], whose mutations caused expanded activation of the JAK2/STAT3 pathway and lymphoid proliferation in vivo and, above all, appeared to coordinate with other genes to promote these disease [23]. The other way round, the studies in the solid tumors is rare. Studies showed that the silencing of LNK in these cells reduced activation of AKT and MAPK signaling and impeded their cell proliferation [24]. The overexpressed LNK in ovarian cancer cells upgraded their proliferation and decreased their cell size, while silenced LNK had different influences [13]. The phosphorylation measures of AKT (upstream of mTOR) and P70-S6 kinase (downstream of mTOR) were each expanded upon LNK overexpression, which suggests that the mTOR pathway is upregulated via LNK in ovarian cancer cells [13, 14, 22, 25]. LNK enhanced the p-AKT and p-MAPK pathways, improved cell adhesion, and moderated cell migration, and advanced the in vivo tumor growth in a murine xenograft model [26, 27], which is in contrast to the detection in hematologic malignancies, and acts as a positive signal transduction modulator in ovarian cancers [13, 14, 28, 29]. On the other hand, there is no relevant literature reporting the role of LNK in TNBC, which is significant for exploring the roles of LNK in TNBC.

Our studies showed that LNK was abnormally expressed in TNBC, thus we can speculate that the LNK may have a certain correlation with TNBC, which indicated that LNK protein may have a certain relationship with the function of TNBC, If indeed LNK functions as a tumor suppressor or promotor in TNBC, possibly it can be a therapeutic target, which can promote us to make progress in TNBC. Therefore, these studies mainly investigated the effects of LNK on TNBC in vivo and in vitro, and further explored the mechanism mediated the effect of LNK on TNBC.

## Materials and methods

### Antibodies and reagents

The following antibodies were used in this study: β-actin (proteintech), LNK (Abcam), p-ERK/ERK (Abcam), p-AKT/AKT (Abcam), p-STAT3/STAT3(CST), p-STAT5/STAT5 (proteintech).

### Cell lines and cell culture

TNBC cell lines MDA-MB-468, MDA-MB-231 (bought by ATCC) which were maintained in DF12 containing 10% fetal bovine serum (FBS). Cells were grown at 37 °C with 5% CO_2_ in humidified air and stable cell line generation.

### Establishment of NOD-SCID mouse breast in situ fat pad model

All animal procedures were performed under standard conditions and in accordance with protocols approved by the Experimental Animal Care Commission at China Pharmaceutical University. We divided our group into non-specific control group (NC), the silenced LNK group 1/2 (SH1/2) and the overexpressed LNK group (OE). The NC, SH1, SH2 and OE MDA-MB-231 cell suspensions (1 × 10^6^) were injected into the breast in situ fat pad and sutured the wound. Tumor growth was directly measured using a caliper. Each group included six mice. When the tumor volume growed into 1000 mm^3^, the mice were sacrificed and the fat pads, tumors, and fat pads containing tumors were removed and embedded in paraffin after 48 h of fixation in buffered formalin.

### Breast cancer tissue chip analysis

The microarray analysis of breast cancer was provided by the Zhongda Hospital affiliated to Southeast University of the hospital. The correlation between LNK and TNBC patients’ gender, age, tumor size, stage, classification and metastasis was analyzed, and the correlation of adverse prognosis was confirmed.

### Construction of LNK stable silencing or overexpressing cell lines

MDA-MB-468 or MDA-MB-231 cells are spotted in dishes, after the cells are attached for 24 h, the cells density reaches 50–60%. The medium was changed to fresh DF12 medium containing 10 μg/ml polybrene, and the lentivirus suspension was separately added and incubated in a 37 °C incubator for 24 h; After non-specific control group (NC) and silent (or) overexpressing LNK virus solution for 48 h, replace it with normal Fresh DF12 medium was further cultured for 24 h; Transfected cells were screened by the addition of puromycin (final concentration 10 μM).

### Apoptosis detection

Adherent cells were collected by trypsin digestion without EDTA. Then wash the cells twice with PBS and use centrifugation at 2000 rpm for 5 min to collect 1–5 × 10^5^ cells; Add 5 μL of 7-AAD dye solution to 50 μL of Binding Buffer and mix. Add the above 7-AAD dye solution to the collected cells and mix; room temperature, protection from light, reaction 5–15 min. After the reaction, add 450 μL of Binding Buffer and mix; Add 1 μL of Annexin V-PE to mix; room temperature, protection from light, reaction for 5 to 15 min;

Flow cytometry detection, excitation wavelength Ex = 488 nm; emission wave length Em = 578 nm, Annexin V-PE orange red fluorescence is recommended to use FL2 channel detection; excitation wavelength Ex = 546 nm; emission wave length Em = 647 nm, 7-AAD Red Fluorescence recommends using FL3 channel detection.

### Cell cycle detection

Wash the cells once with PBS (centrifugation 2000 rpm, 5 min), collect and adjust the cell concentration to 1 × 10^6^/mL, and take 1 mL of single cell suspension; After preparing the single cell suspension, the supernatant is removed, 500 μL of an equal volume of 70% cold ethanol is added to the cells, and fixed at 4 °C overnight, and the fixing solution is washed away with PBS before dyeing; Add 100 μL of RNAaseA in a 37 °C water bath for 30 min; further add 400 μL PI staining and mix, avoiding light for 30 min at 4 °C; On-machine detection, recording red fluorescence at 488 nm wave length of excitation light.

### Transwell migration experiment

The SH1/2 and NC group cells of MDA-MB-231/MDA-MB-468 and the cells of OE and NC groups were collected and counted; the Transwell migration chamber was placed in a 24-well plate, and added 500 μL of medium containing 10% FBS to the outer chamber. Then 200 μL of cell suspension containing 5 × 10^4^ cells was added to the inner chamber, and after 12 h of incubation in the incubator, the stains I in the Diff stain solution box was fixed for 10 min, and the stains II and III were stained for 10 min, respectively, and washed. Photographed on a microscope and used Image J software to calculate the number of migrated cells.

### Wound healing experiment

The SH1/SH2 and NC group cells of MDA-MB-231/MDA-MB-468 and the cells of OE and NC groups were collected, counted, and added to a six-well plate at 1 × 10^6^ cells per well; the incubator was placed overnight until the cells were attached. After that, use the sterilized white gun head to draw the word “well” in each hole; wash it in PBS twice, remove the residual cell debris; place it in the incubator for constant temperature culture; shoot the same “cross” area of the cells at different time periods, observe cell scratch healing ability.

### Plateclone formation experiment

The SH1/SH2 and NC group cells of MDA-MB-231/MDA-MB-468 and the OE and NC group cells were collected, digested, centrifuged, and the cell suspension was counted and diluted to 1–2 × 10^5^ cells/mL. A corresponding volume of the cell suspension was taken in complete medium, and after pipetting and mixing, the cell suspension was added to a 6-well plate. 2 mL of cell suspension per well contains 1000 cells. Incubation was continued in the incubator until at least 50 cells in a single cell mass, and the culture was terminated. The 6-well plate was removed; the supernatant was discarded, and washed twice with PBS. Add 1 mL of the chamber R1 stain solution to each well and fix it at room temperature for 15 min. After the end, the fixing solution was removed, and 1 mL of crystal violet dye solution was added to each well, and the mixture was allowed to stand at room temperature for 15–20 min. Finally, rinse the bottom of the board with a small amount of tap water to wash away the excess crystal violet. Take pictures after the 6-well plate is dry.

### Edu proliferation experiment

The SH1/SH2 and NC group cells of MDA-MB-231/MDA-MB-468 and the cells of OE and NC groups were collected, digested, centrifuged, and the cell suspension was counted and diluted to 5 × 10^4^ cells/mL to be seeded in 12 wells. Plate cell slides were placed in a 37 °C constant temperature, 5% CO_2_ incubator for 24 h; the previous medium was discarded, fresh complete medium containing 40 μM EDU was added, and cultured at 37 °C constant temperature, 5% CO_2_ Incubate the box for 2 h, and mark the proliferating cells with EDU; remove the medium, add 1 mL of 4% neutral formaldehyde per well, and incubate for 15 min at room temperature; remove the fixative and prepare 3% BSA in 1 mL PBS per well. Wash the cells twice with washing solution; remove the washing solution, add 0.5 mL Triton X-100 in 1 mL PBS per well, incubate for 20 min at room temperature; remove the permeation solution, and wash the 3% BSA solution in 1 mL PBS per well. Wash twice; remove the washing solution; add 0.5 mL of 1× Click-iT reaction mixture to each well, gently shake the plate to ensure that the reaction mixture is evenly covered with the slides, incubate for 30 min at room temperature in the dark; remove the reaction mixture and add to each well. The cells were washed twice with 3% BSA in 1 mL PBS. Discard the old washing solution, wash once with 1 mL of PBS, remove the washing solution; dilute Hoechst 33342 staining solution with PBS to a final concentration of 5 μg/mL, and add 1 mL of 1× Hoechst 3342 dilution to each well. Incubate for 30 min at room temperature on a shaker in the dark; remove the Hoechst 3342 solution and wash once with 1 mL of PBS per well to remove the wash. Fix the cells and take a photo under a fluorescence microscope.

### Western blot assay

Cellular or tissue proteins were extracted and analyzed by western blot. The total cell lysates were extracted from the cells, adding phosphatase and prote-ase inhibitors. Total proteins (20 μg) were separated via 8–15% SDS-PAGE and transferred onto PVDF membrane (Millipore, USA). Next, the membranes were blocked with 5% BSA and incubated with primary anti-bodies (dilution in 5% BSA-TBST) for overnight at 4 °C. Then, probed it with secondary antibody for 1 h at room temperature. Subsequently, the expression of the target proteins was detected by Chemiluminescent HRP Sub-strate (Millipore, USA).

### Quantitative real time PCR

The total cellular RNA was isolated using the TRIzol Reagent (Vazyme, Nanjing, China) and reverse transcribed with the HiScript QRT SuperMix for qPCR (Vazyme). The mRNA levels were measured using the SYBR Green master mix (Vazyme).

## Results

### High expression of LNK in triple-negative breast cancer

**Fig. 1:**
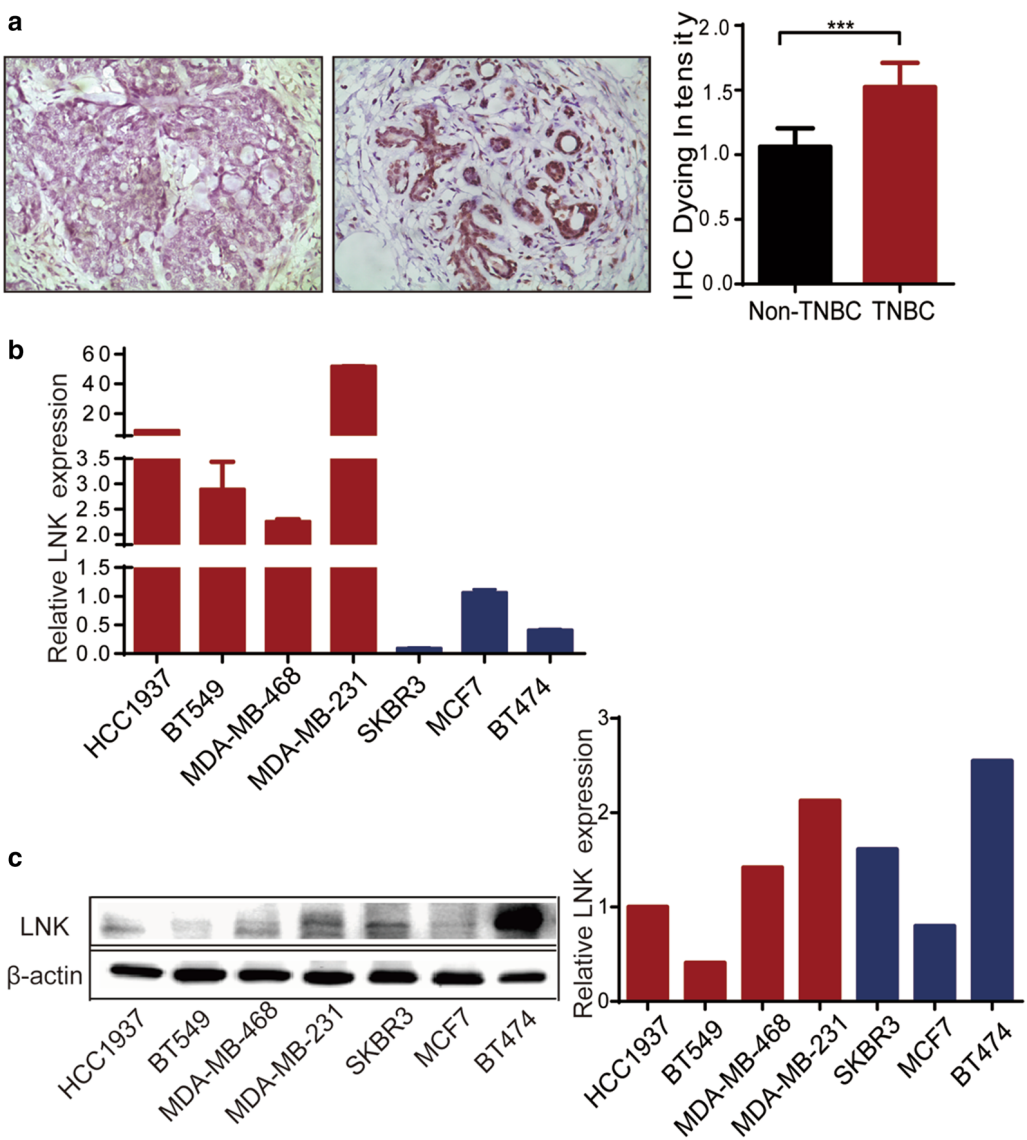
Expression of LNK in breast cancer cell lines and tissues. **a** Expression of LNK in tissue chips of TNBC and non-triple negative breast cancer, n = 8. **b** RT-PCR was used to investigate the expression of LNK in different breast cancer cell lines, which included MCF7, SKBR3, BT474, MDA-MB-468, MDA-MB-231, HCC1937, BT549. **c** Western blots was used to investigate the expression of LNK in different breast cancer cell lines, which included MCF7, SKBR3, BT474, MDA-MB-468, MDA-MB-231, HCC1937, BT549

Previous studies showed that overexpression of LNK inhibited cell growth and caused cell death in many leukemia cell lines, but similar experiments performed in several solid tumor cell lines had little effect on their proliferation, suggesting that LNK might have a different role in solid tumor cells compared to those of the hematopoietic system. Clinical tissue chips studies have indicated that LNK is abnormally expressed in breast cancer, which indicates that LNK may have a certain effect on breast cancer. We firstly compared the expression of LNK in non-TNBC to TNBC and found that LNK was higher expression in triple-negative breast cancer than in non-negative breast cancer (Fig. 1a). Subsequently, we investigated the expression of LNK in several breast cancer cell lines by RT-PCR and western blots. Unexpectedly, we found that LNK were significantly upregulated (Fig. 1b, c) during TNBC cell lines (MDA-MB-468, MDA-MB-231, HCC1937, BT549), compared to those of non-triple negative breast cancer cell lines (MCF7, SKBR3, BT474), when the cells were cultivated to complete medium containing 10% FBS. Therefore, in the following studies, we mainly investigate the roles of TNBC cell lines (MDA-MB-468 and MDA-MB-231).

### LNK did not play roles on cell cycle and apoptosis of TNBC

**Fig. 2:**
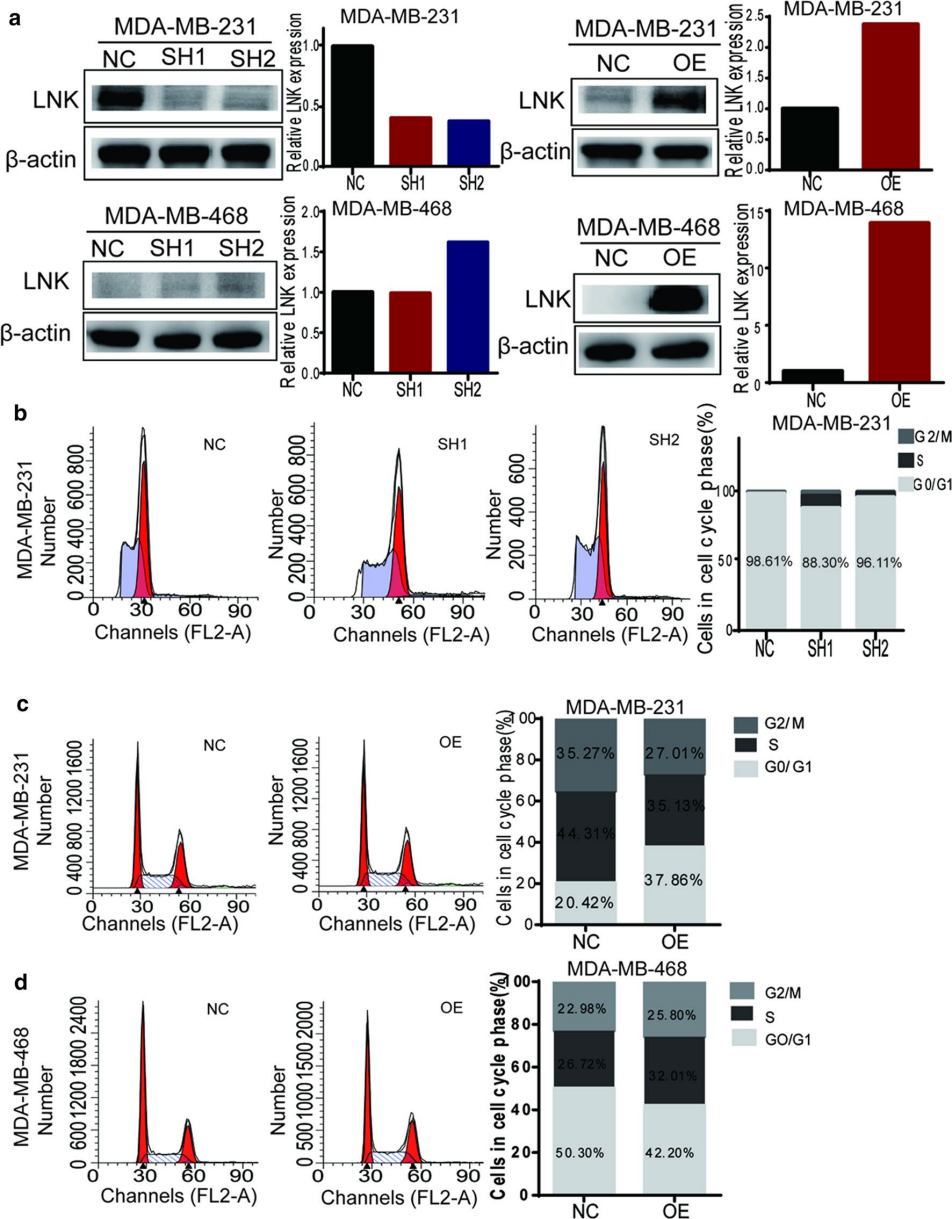
The effect of LNK on cell cycle in TNBC cells. **a** According to the expression of LNK at the protein level, breast cancer cells MDA-MB-231 and MDA-MB-468 with expression of LNK were selected to construct LNK stably silenced and overexpressed cell lines, and the efficiency was verified by Western blot. **b**–**d** The effect of silencing and over-expressing LNK on the cell cycle of TNBC cell lines MDA-MB-231 and MDA-MB-468 were investigated by using cell cycle detection kit

To explore the function of LNK in TNBC, we choose MDA-MB-231 and MDA-MB-468 to construct LNK stably silenced and overexpressed cell lines, which were verified by Western blot (Fig. 2a). In addition, MDA-MB-231 had a significant silencing efficiency, while MDA-MB-468 had no obvious silencing efficiency (Fig. 2a). This is mainly related to the low expression of LNK in MDA-MB-468 cells (Fig. 1b, c). Therefore, to explore the functional importance of this finding in TNBC, we mainly studied both forced-expression and silencing of LNK in MDA-MB-231 and MDA-MB-468 for the subsequent experiments.

**Fig. 3:**
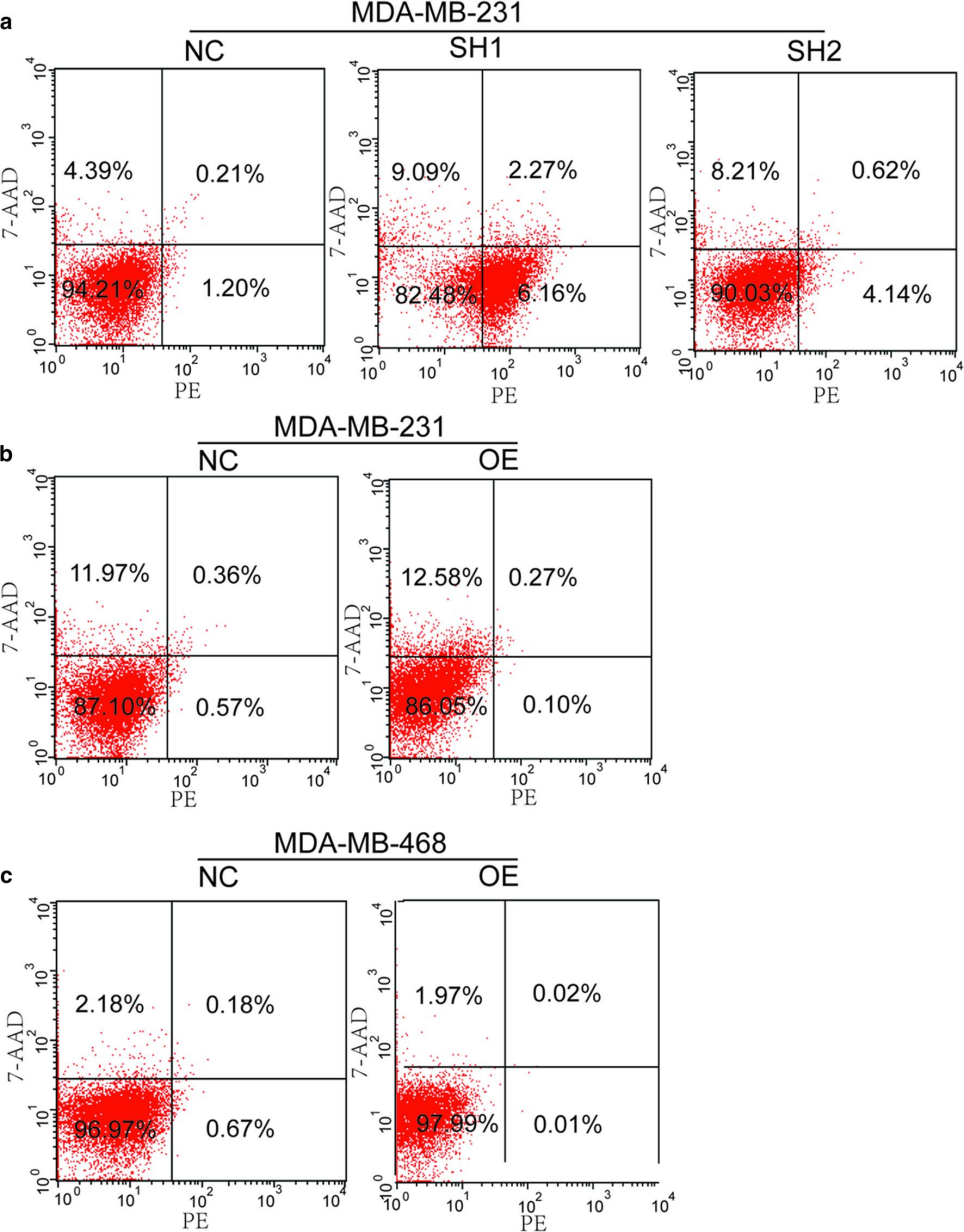
The effect of LNK on cell apoptosis in TNBC cells lines. **a**–**c** The effect of silencing and over-expressing LNK on the cell cycle of TNBC cell lines MDA-MB-231 and MDA-MB-468 were investigated by using Apoptosis kit

Firstly, we examined the effect of silencing and over-expressing LNK on the cell cycle of breast cancer by PI staining. Our study shows that the silencing and over-expressing of LNK had no significant effect on cell cycle in MDA-MB-231 (Fig. 2b–d). Then, we investigated the effect of LNK on cell apoptosis in MDA-MB-231 cells and MDA-MB-468 cells, the results showed that the silencing and overexpressing of LNK had no significant effect on apoptosis in MDA-MB-231 cells or MDA-MB-468 cells (Fig. 3a–c). Therefore, these results suggested LNK have no effect on cell cycle and apoptosis of breast cancer cells, which is similar to the previous studies in ovarian cancer [15].

### LNK promoted the proliferation and migration ability in vitro

**Fig. 4:**
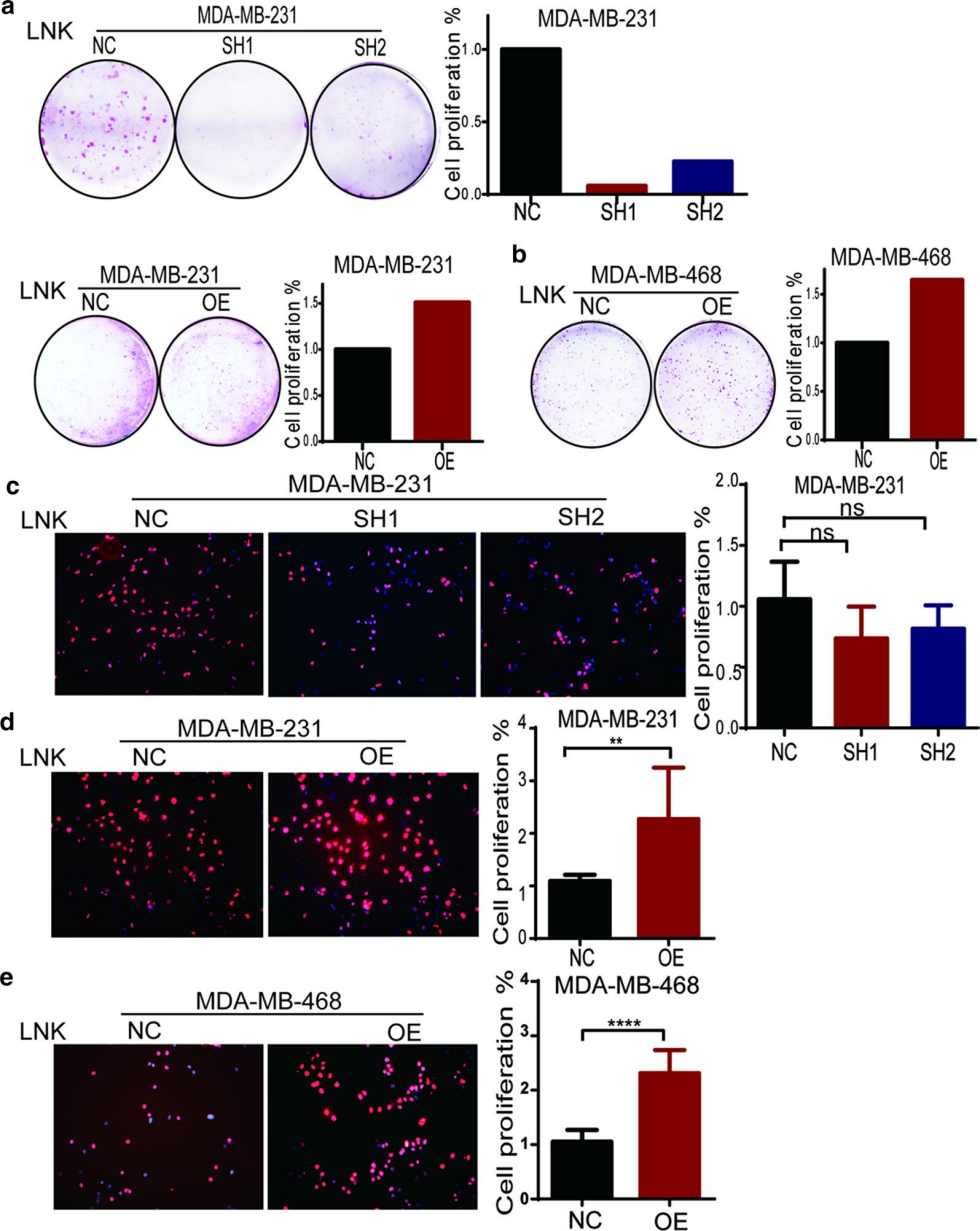
Effect of silencing or over-expression of LNK on proliferation of breast cancer cells. **a**, **b** Clonal formation assay was used to investigate the effect of silencing and overexpression of LNK on proliferation of breast cancer cells. **c**–**e** Edu staining assay was used to investigate the effect of silencing and overexpression of LNK on proliferation of breast cancer cells

In order to verify the effect of LNK on proliferation of TNBC cells, we performed cell colony formation assay and Edu staining assay. The results showed that numbers of TNBC cells in the medium were significantly inhibited after silencing of LNK in MDA-MB-231, which indicated that LNK might affect the proliferation of TNBC cells (Fig. 4a). On the other hand, when the MDA-MB-231 and MDA-MB-468 were forced expressing LNK, the number of TNBC cells were significantly increased (Fig. 4a, b), which reversely showed that LNK can promote the growth of TNBC cells. Therefore, the above results preliminarily suggested that LNK could promote TNBC cells to proliferate.

Furtherly, we used Edu staining to verify the proliferative role of LNK in TNBC. The results showed that the MDA-MB-231 was forced silenced LNK, the number of breast cancer cells in the medium were reduced, and on the other hand, the MDA-MB-468 were forced expressing LNK, the number of breast cancer cells were significantly increased (Fig. 4c–e), which was consistent with the results of the colony formation assay, which furtherly confirmed that LNK can promote TNBC proliferation.

**Fig. 5:**
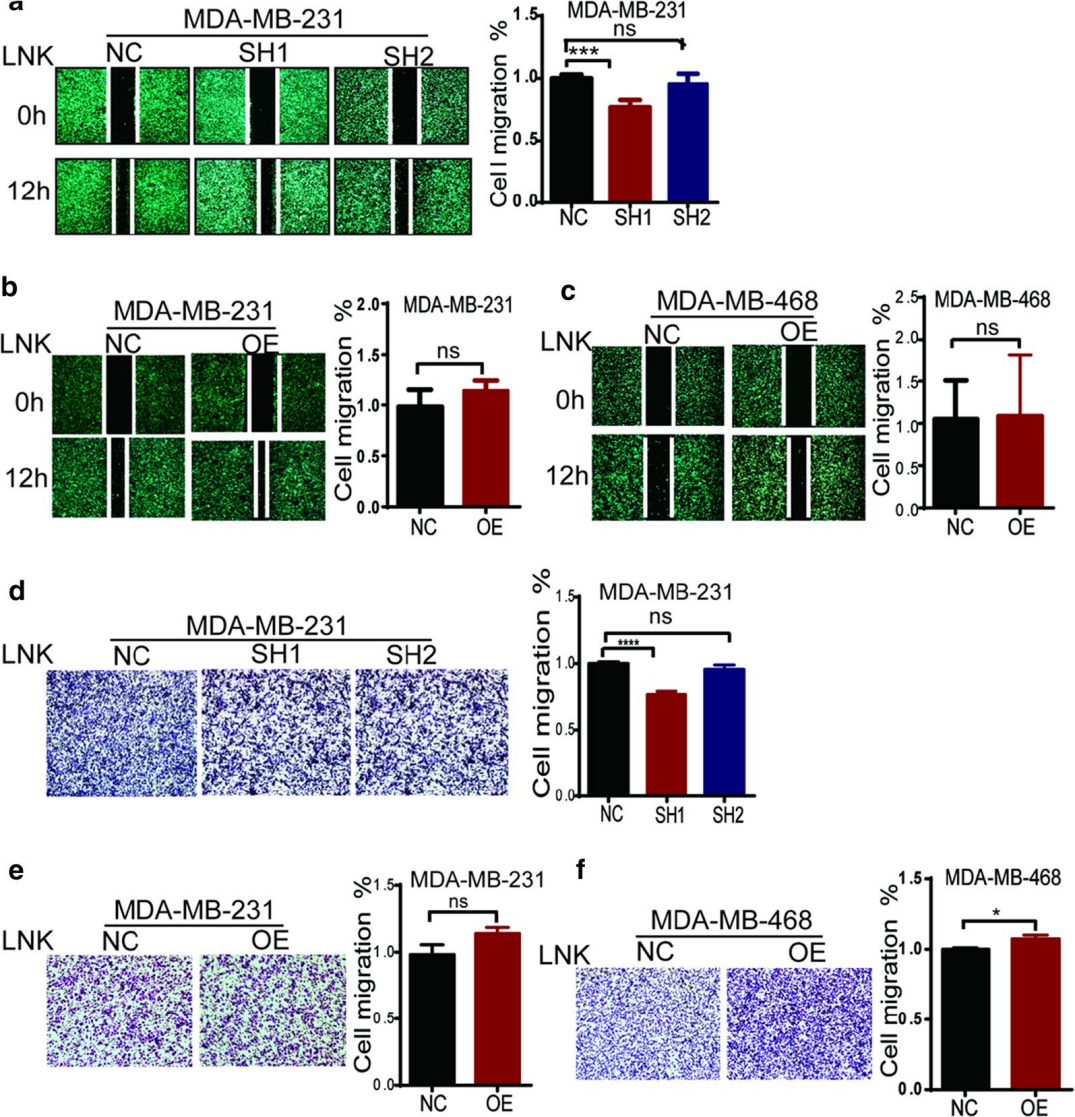
Effect of silencing or over-expression of LNK on Cell migration of TNBC cells. **a**, **b** Effect of silencing and over-expressing LNK on cell migration of TNBC using scratch test. **c**–**e** effect of silencing and over-expressing LNK on cell cycle of breast cancer using Trans well migration experiment

Cell migration was measured by using the wound healing assay (Fig. 5a–c). After 12 h of the wound scratch, silencing LNK MDA-MB-231 cells maintained a nearly intact gap. In contrast, the control cells nearly closed the healing gap, indicating that LNK may indeed play a role in regulation of cell migration. On the other hand, overexpressing LNK MDA-MB-231 cells closed the healing gap nearly, compared to the control group, which indicated that LNK may promote TNBC cells to migrate. But the forced-expressing LNK MDA-MB-468 cells didn’t show the similar results (Fig. 5c). In addition, the transwell experiments found that silencing LNK of MDA-MB-231(Fig. 5d, e) migrated significantly less than the control group. Similarly overexpressing LNK of MDA-MB-231 (Fig. 5e) and MDA-MB-468 (Fig. 5f) migrated more. It indicates that the LNK may have a function of promoting cell migration in TNBC cells, which is different from the studies in the ovarian cancer [14].

### LNK promotes tumor growth and migration in an in vivo murine xenograft model

To verify the effect of LNK on TNBC in vivo, we investigated the effect of LNK on TNBC growth via the in vivo xenograft tumor experiment. Firstly we used NOD-SCID female mice to inoculate MDA-MB-231-NC, MDA-MB-231-SH1/2 and MDA-MB-231-OE cells in situ with a mammary fat pad. And when the tumor volume reached 100 mm^3^, we measured the tumor volume three times a week, observed the activity of the mice and recorded the death of the mice. When the tumor volume reached 1000 mm^3^, the mice were sacrificed and we observed the Lymph node and lung migration of the xenograft tumor mice in vivo.

**Fig. 6:**
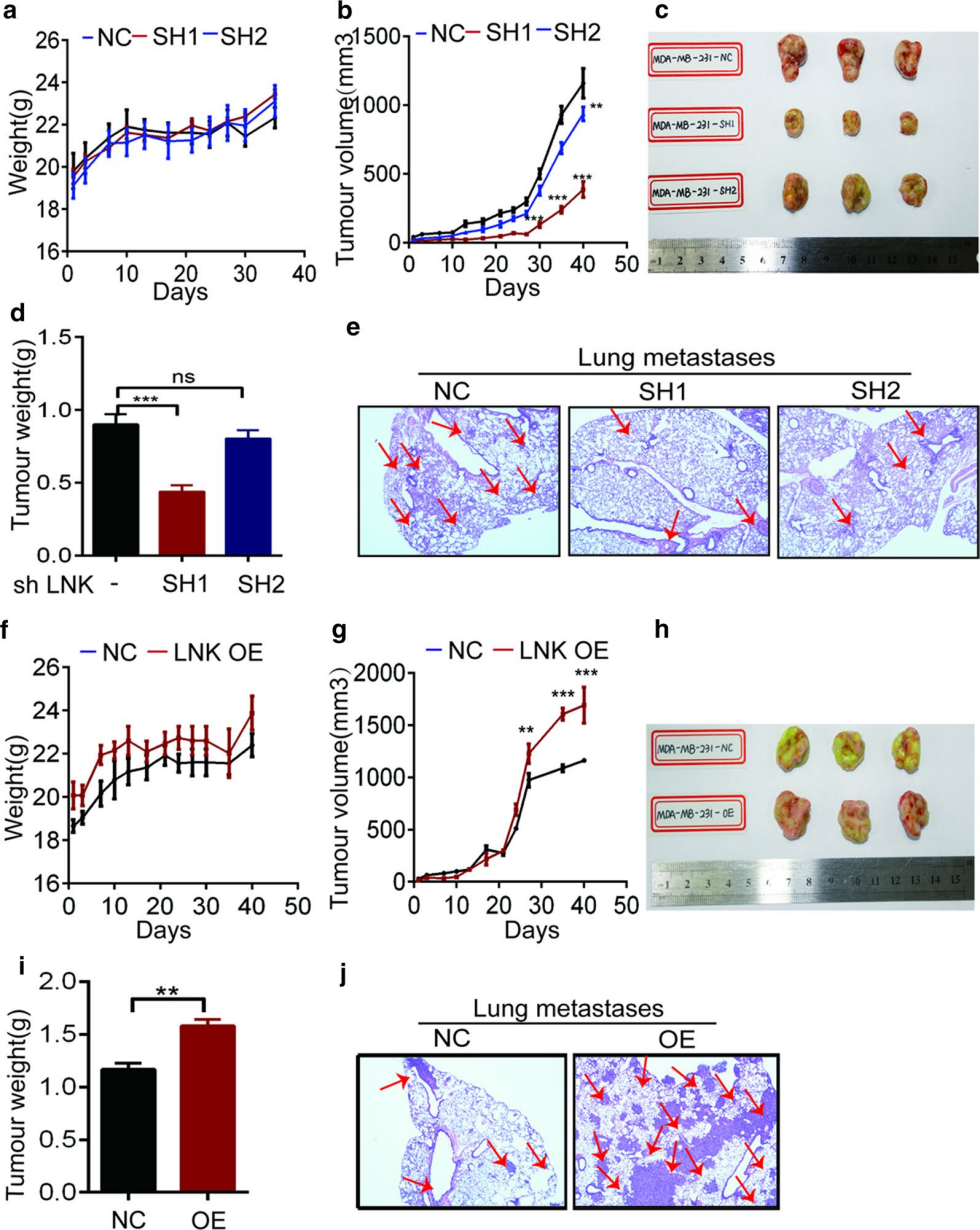
Effects of forced expression or silencing of LNK on tumor growth and migration in vitro. **a**, **f** Effects of either forced expression or silencing of LN on mouse weight. **b**, **g** Effects of either forced expression or silencing of LNK on effect on mouse tumor volume. **c**, **h** The status of LNK tumor status which it is in vitro forced expression or silencing of LNK. **d**, **i** In vitro effects of either forced expression or silencing of LNK on effect of mouse tumor weight. **e**, **j** Immunohistochemistry of lung metastasis in LNK knockdown and surgically transplanted tumor mice

Compared to the non-specific control (NC) group, the knockdown (SH1/2) and the overexpression (OE) group had no significant effect on the body weight of the mice (Fig. 6a, f). However, compared to the NC group, the tumor volume of the knockdown group was significantly reduced (Fig. 6b); while the OE group tumor growth rate was lower than the NC group in the first 20 days. And 20 days later, the growth rate is accelerated, which resulted in the OE group tumor volume becoming bigger than the NC group (Fig. 6g). And the mice tumor figure of the knockdown and overexpression group showed the similar results (Fig. 6c, h). Our results showed that lung metastasis was significantly less in the knockdown group than NC group and the tumor weight of the knockdown group was significantly lower than that of the control group (Fig. 6d, e), and at the same time, the lung metastasis and the tumor weight of the OE group was significantly increased (Fig. 6i, j). The above results indicate that the LNK can also promote the proliferation and migration of breast cancer cells in vivo, which is consistent with our results in vitro.

### Mechanism of LNK promoting breast cancer proliferation or migration

**Fig. 7:**
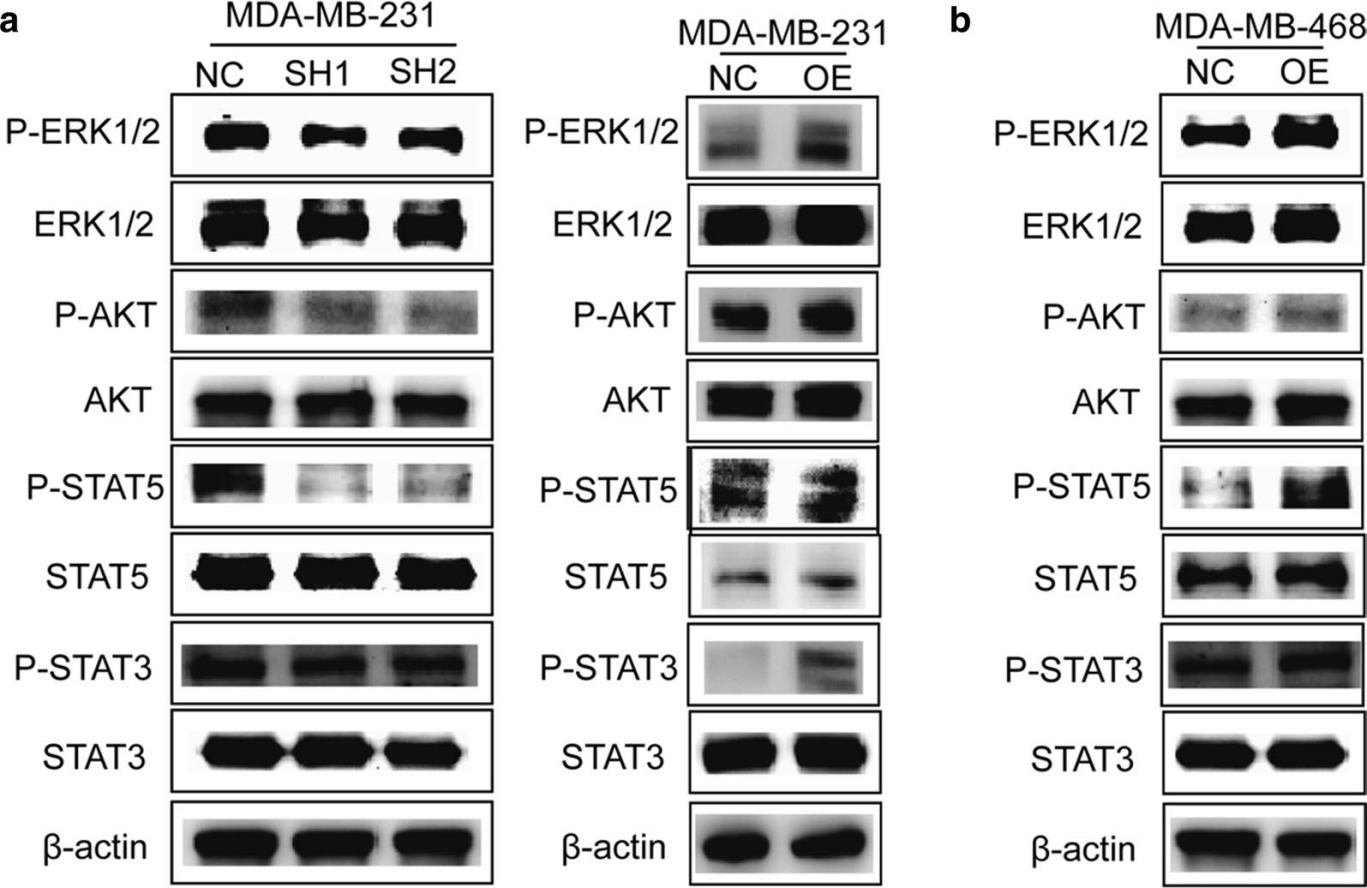
LNK upregulates mitogen signaling pathways in TNBC cells. **a**, **b** Western blot analysis of molecular mechanisms which were related to silencing and over-expressing of LNK on TNBC cells

Previous studies showed that LNK interacts closely with JAK2-STAT, PI3K/AKT, and ERK1/2 signaling pathway-related proteins [21, 30, 31]. It is speculated that LNK may affect the proliferation and migration of breast cancer cells through these key signaling pathways. Therefore, we verify this signaling pathways through the Western blot analysis (Fig. 7a, b) and found that when MDA-MB-231 silenced LNK, p-ERK1/2 and p-STAT5 signaling pathways were significantly inhibited, while p-AKT and p-STAT3 were also inhibited to some extent (Fig. 7a). Conversely, when MDA-MB-231 overexpressed LNK, the p-ERK1/2 and p-STAT5 pathways were significantly activated (Fig. 7a). At the same time, MDA-MB-468 cells overexpressed LNK, p-ERK1/2 and p-STAT3 were significantly activated (Fig. 7b). In addition, the p-AKT pathway was slightly activated, while the p-STAT5 pathway was not significantly altered (Fig. 7b). These results confirmed that LNK mainly activates the p-ERK1/2 pathway, and in addition, have a certain degree of activation with the p-STAT5, p-AKT and p-STAT3 pathways, thereby promoting breast cancer cell proliferation and migration ability, which is similar to the studies in Ovarian cancer [14].

## Discussion

LNK (SH2B3) is an adaptor protein studied extensively in normal and malignant hematopoietic cells, which plays an important role in Multiple blood diseases, such as Fanconi anemia (FA), Acute lymphoblastic leukemia (ALL), Aortic dissection (AD) and so on [31–33]. And research also shows that LNK mutations have recently been found in patients with myeloproliferative neoplasms (MPNs), early T cell acute lymphoblastic leukemia, Ph-like acute lymphoblastic leukemia, B-precursor acute lymphoblastic leukemia, and Down syndrome-related myeloid disorders [29, 31, 32, 34, 35]. Though the clinical sample chip analysis, we found that the LNK is abnormally expressed in breast cancer, which may play a different role in breast cancer. Furtherly, our research showed that the expression of LNK in TNBC is higher than that in breast cancer. Besides, previous studies showed that LNK has few studies on solid tumors except for ovarian cancer [14]. On the other hand, clinical studies proved that LNK has a high surface state in TNBC which prompted us to study whether LNK also has a certain functional relevance to TNBC.

It is important to realize that our groups and others have previously demonstrated that LNK might play an alternate role in solid tumor cells in hematopoietic and lymphoid cancers [36, 37], while it showed few effects on proliferation and migration in some solid tumor cell lines [35], and regarded as a negative inhibitor in tumor [38]. In this study, we found that when LNK was over-expressed in MAD-MB-468 and MDA-MB-231 cells, it makes the proliferative capacity significantly enhanced. On the contrary, we found that when the LNK was silenced, the TNBC proliferative capacity significantly reduced. It was initially proved that the LNK may have a certain promoted effect on the proliferation of TNBC cells in vitro. It was initially showed that LNK may have a certain promoted effect on the proliferation of TNBC cells, which is similar to the ovarian cancer [14] but different from Multiple blood diseases, like, Fanconi anemia (FA), Acute lymphoblastic leukemia (ALL), Aortic dissection (AD) and so on [21, 31–33].

Furtherly, Previous studies have shown that LNK affects cell adhesion, ECM interaction and so on, which is consistent with the result that overexpression of LNK inhibited cell migration in normal endothelial cells [24, 37]. Additionally, LNK was recently found to play an important role in the regulation of focal adhesion complexes [14, 34]. In this study, we found that the over-expressing LNK can promote tumor migration, which is contrary to previous studies. When we silenced LNK, we found that the migration ability of breast cancer cells was opposite to that of ovarian cancer [14]. In this study, we found that over-expression of LNK can promote breast cancer migration. Our research also showed that LNK had no significant effect on the cell cycle and apoptosis of TNBC, which has also not been reported in other cancer.

The in vivo xenograft experiments indicated that LNK can promote cell growth and generate bigger tumor. Then, why does LNK appear different in leukemia cells verses TNBC cell? LNK does not have enzymatic activity, whose function is totally dependent on its binding partners [36, 38]. And we also find that the other two members [SH2B1 and APS (SH2B2)] of this family of proteins share similar sequence homologies in leukemia cells, whose stimulatory and inhibitory roles also appear to be cell-type and pathway dependent [35, 39]. Many hematopoietic malignancies including MPN, act as classical “activated kinase diseases” driven by a mutant activated receptor tyrosine kinase (e.g. FLT3-ITD, mutant c-KIT) or the downstream kinase. Besides participating in cell adhesion and ECM-interaction, pathway analysis suggested a possible role of LNK in the JAK2-STAT and p-ERK1/2 signaling pathways. LNK is a lymphocyte-specific adaptor protein that plays, which has an essential role part in the JAK-STAT signaling pathway via negative regulation of normal hematopoiesis. LNK is capable of coordinating with additional oncogenic pathways likes the JAK/STAT signaling, which suggests recommending a suppressor role for LNK in MPN development [30, 39]. LNK was appeared to tie both wild-type and MPN mutant types of the MPL receptor (MPLW515L) and to antagonize the activation of JAK2, STAT3, ERK, and AKT [30, 36]. Our study proved that when TNBC was silenced LNK, p-ERK1/2 and p-STAT5 signaling pathways were significantly inhibited, and p-AKT and p-STAT3 were also inhibited, which confirmed that LNK mainly activates the p-ERK1/2 pathway. In addition, the p-STAT5, p-AKT and p-STAT3 pathways also have a certain degree of activation, thereby promoting breast cancer cell proliferation and migration ability. While, in ovarian cancers LNK mainly activated the p-AKT and p-MAPK pathways, improved cell adhesion, moderated cell migration, and advanced in vivo tumor growth in a murine xenograft model [14], which is similar to TNBC. In contrast to detection in hematologic malignancies, the adaptor protein LNK acts as a positive signal transduction modulator in solid tumors [14].

## Conclusion

In summary, our data firstly identified several unique functions of LNK in TNBC cells. LNK augmented the p-AKT, p-MAPK and ERK1/2 pathways, promoted cell migration, enhanced cell adhesion, and promoted the in vivo tumor growth and migration in vivo xenograft tumor model. Our results suggest that the adaptor protein LNK acts as a positive signal transduction modulator in TNBC which is similar to the ovarian cancer, and it is in contrast to the findings in hematologic malignancies, We believe that our observations are novel and open a new area of inquiry for this important adaptor protein, which may be regarded as a new drug targets in the following studies.

## Data Availability

The datasets used and/or analyzed during the current study are available from the corresponding author on reasonable request.

## Abbreviations

TNBC: Triple negative breast cancers
ER: Estrogen
PR: Progesterone
HER2: Human epidermal growth factor receptor-2
PH: Pleckstrin homology
SCF: Stem cell factor
MPL: Myeloproliferative leukemia protein
EPO: Erythropoietin
PDGF: Platelet-derived growth factor
TNF: Tumor necrosis factor
MPN: Myeloproliferative neoplasms
RT-PCR: Quantitative real time PCR

## References

[1] Chang Yao-Yin, Kuo Wen-Hung (2015). Deregulated microRNAs in triple-negative breast cancer revealed by deep sequencing. Mol Cancer.

[2] Al-Mahmood Sumayah, Sapiezynski Justin (2018). Metastatic and triple-negative breast cancer: challenges and treatment options. Drug Deliv Transl Res.

[3] Maxmen A (2012). The hard facts. Nature.

[4] Andrea M, Rodney J (2016). miRNAs and other epigenetic changes as biomarkers in triple negative breast cancer. Mol Sci..

[5] Cheng Ying, Chikwava Kudakwashe (2016). LNK/SH2B3 regulates IL-7 receptor signaling in normal and malignant B-prgenitors. J Clin Investig.

[6] Nicola F, Felipe CG (2016). Genetic events in the progression of adenoid cystic carcinoma of the breast to high-grade triple-negative breast cancer. Mod Pathol.

[7] Palma Giuseppe, Frasc Giuseppe (2015). Triple negative breast cancer: looking for the missing link between biology and treatments. Oncotarget..

[8] Boyle P (2012). Triple-negative breast cancer: epidemiological considerations and recommendations. Ann Oncol.

[9] Xu H, Eirew P, Mullaly SC, Aparicio S (2014). The omics of triple-negative breast cancers. Clin Chem.

[10] Li Z, Ren M, Tian J, Jiang S, Liu Y, Zhang L, Wang Z, Song Q, Liu C, Wu T (2015). The differences in ultrasound and clinic pathological features between basal-like and normal like subtypes of triple negative breast cancer. PLoS ONE.

[11] Velazquez L, Cheng AM, Fleming HE, Furlonger C, Vesely S, Bernstein A (2002). Cytokine signaling and hematopoietic homeostasis are disrupted in Lnk-deficient mice. J Exp Med.

[12] Takaki S, Morita H, Tezuka Y, Takatsu K (2002). Enhanced hematopoiesis by hematopoietic progenitor cells lacking intracellular adaptor protein. Lnk. J Exp Med..

[13] Wang Wei, Tang Yang (2016). LNK/SH2B3 loss of function promotes atherosclerosis and thrombosis. Circ Res.

[14] Ding Ling-Wen, Sun Qiao-Yang (2015). LNK (SH2B3): paradoxical effects in ovarian cancer. Oncogene.

[15] Balcerek Joanna, Jiang Jing (2018). Lnk/Sh2b3 deficiency restores hematopoietic stem cell function and genome integrity in Fancd2 deficient Fanconi anemia. Nat Commun.

[16] Fanny L, Arina K (2018). LNK deficiency promotes acute aortic dissection and rupture. JCI Insight..

[17] Jun HL, Sang HL (2016). Lnk is an important modulator of insulin-like growth factor-1/Akt/peroxisome proliferator-activated receptor-gamma axis during adipogenesis of mesenchymal stem cells. Korean J Physiol Pharmacol..

[18] David J, Olivos I, Marta A (2017). Lnk Deficiency leads to TPO-mediated osteoclastogenesis and increased bone mass phenotype. J Cell Biochem.

[19] Gery S, Cao Q, Gueller S, Xing H, Tefferi A, Koeffler HP (2009). Lnk inhibits myeloproliferative disorder-associated JAK2 mutant, JAK2V617F. J Leukoc Biol.

[20] Bersenev A, Wu C, Balcerek J, Jing J, Kundu M, Blobel GA (2010). Lnk constrains myeloproliferative diseases in mice. J Clin Invest..

[21] Devallière J, Charreau B (2011). The adaptor Lnk (SH2B3): an emerging regulator in vascular cells and a link between immune and inflammatory signaling. Biochem Pharmacol.

[22] Flister MJ, Hoffman MJ, Lemke A, Prisco SZ, Rudemiller N, O’Meara CC, Tsaih SW, Moreno C, Geurts AM, Lazar J, Adhikari N, Hall JL, Jacob HJ (2015). SH2B3 is a genetic determinant of cardiac inflammation and fibrosis. Circ Cardiovasc Genet..

[23] Velazquez L (2012). The Lnk adaptor protein: a key regulator of normal and pathological hematopoiesis. Arch Immunol Ther Exp (Warsz).

[24] Devalliere J, Chatelais M, Fitau J, Gerard N, Hulin P, Velazquez L (2012). LNK (SH2B3) is a key regulator of integrin signaling in endothelial cells and targets alpha-parvin to control cell adhesion and migration. FASEB J..

[25] Gery S, Gueller S, Nowak V, Sohn J, Hofmann WK, Koeffler HP (2009). Expression of the adaptor protein Lnk in leukemia cells. Exp Hematol.

[26] Matsumoto T, Ii M, Nishimura H, Shoji T (2010). Lnk-dependent axis of SCF-cKit signal for osteogenesis in bone fracture healing. J Exp Med.

[27] Saleh MA, McMaster WG, Wu J (2015). Lymphocyte adaptor protein LNK deficiency exacerbates hypertension and endorgan inflammation. J Clin Invest..

[28] Baran-Marszak Fanny, Magdoud Hajer, Desterke Christophe (2010). Expression level and differential JAK2-V617F-binding of the adaptor protein Lnk regulates JAK2-mediated signals in myeloproliferative neoplasms. Blood.

[29] Lanning NJ, Su HW, Argetsinger LS, Carter-Su C (2011). Identification of SH2B1 beta as a focal adhesion protein that regulates focal adhesion size and number. J Cell Sci.

[30] Lee SH, Lee KB, Lee JH, Kang S, Kim HG, Asahara T, Kwon SM (2015). Selective interference targeting of Lnk in umbilical cord-derived late endothelial progenitor cells improves vascular repair, following hind limb ischemic injury, via regulation of JAK2/STAT3 signaling. Stem Cells..

[31] Cheng Ying, Chikwava Kudakwashe, Chao Wu (2016). LNK/SH2B3 regulates IL-7 receptor signaling in normal and malignant B-progenitors. J Clin Invest..

[32] Laroumanie Fanny, Korneva Arina, Bersi Matthew R (2018). LNK deficiency promotes acute aortic dissection and rupture. J CI Insight..

[33] Balcerek Joanna, Jiang Jing, Li Yang (2018). Lnk/Sh2b3 deficiency restores hematopoietic stem cell function and genome integrity in Fancd2 deficient Fanconi anemia. Nature Communication..

[34] Nishi M, Werner ED, Oh BC, Frantz JD, Dhe-Paganon S, Hansen L (2005). Kinase activation through dimerization by human SH2-B. Mol Cell Biol.

[35] Gery S, Koeffler HP (2013). Role of the adaptor protein LNK in normal and malignant hematopoiesis. Oncogene.

[36] Ahmed Z, Pillay TS (2003). Adapter protein with a pleckstrin homology (PH) and an Src homology 2 (SH2) domain (APS) and SH2-B enhance insulin-receptor autophosphorylation, extracellular signal-regulated kinase and phosphoinositide 3-kinase-dependent signalling. Biochem J..

[37] Perez-Garcia A, Ambesi-Impiombato A, Hadler M, Rigo I, Leduc CA, Kelly K, Jalas C, Paietta E, Racevskis J, Rowe JM, Tallman MS, Paganin M, Basso G, Tong W, Chung WK, Ferrando AA (2013). Genetic loss of SH2B3 in acute lymphoblastic leukemia. Blood.

[38] Qian X, Ginty DD (2001). SH2-B and APS are multimeric adapters that augment TrkA signaling. Mol Cell Biol.

[39] Bersenev A, Wu C, Balcerek J, Jing J, Kundu M, Blobel GA, Chikwava KR, Tong W (2010). Lnk constrains myeloproliferative diseases in mice. J Clin Investig.

